# Supplementary figures and images for: Association Analysis of Single-Cell RNA Sequencing and Proteomics Reveals a Vital Role of Ca2+ Signaling in the Determination of Skeletal Muscle Development Potential
Source: Cells. 2020 Apr 22;9(4):1045. doi: 10.3390/cells9041045 (PMC7225978; doi:10.3390/cells9041045)

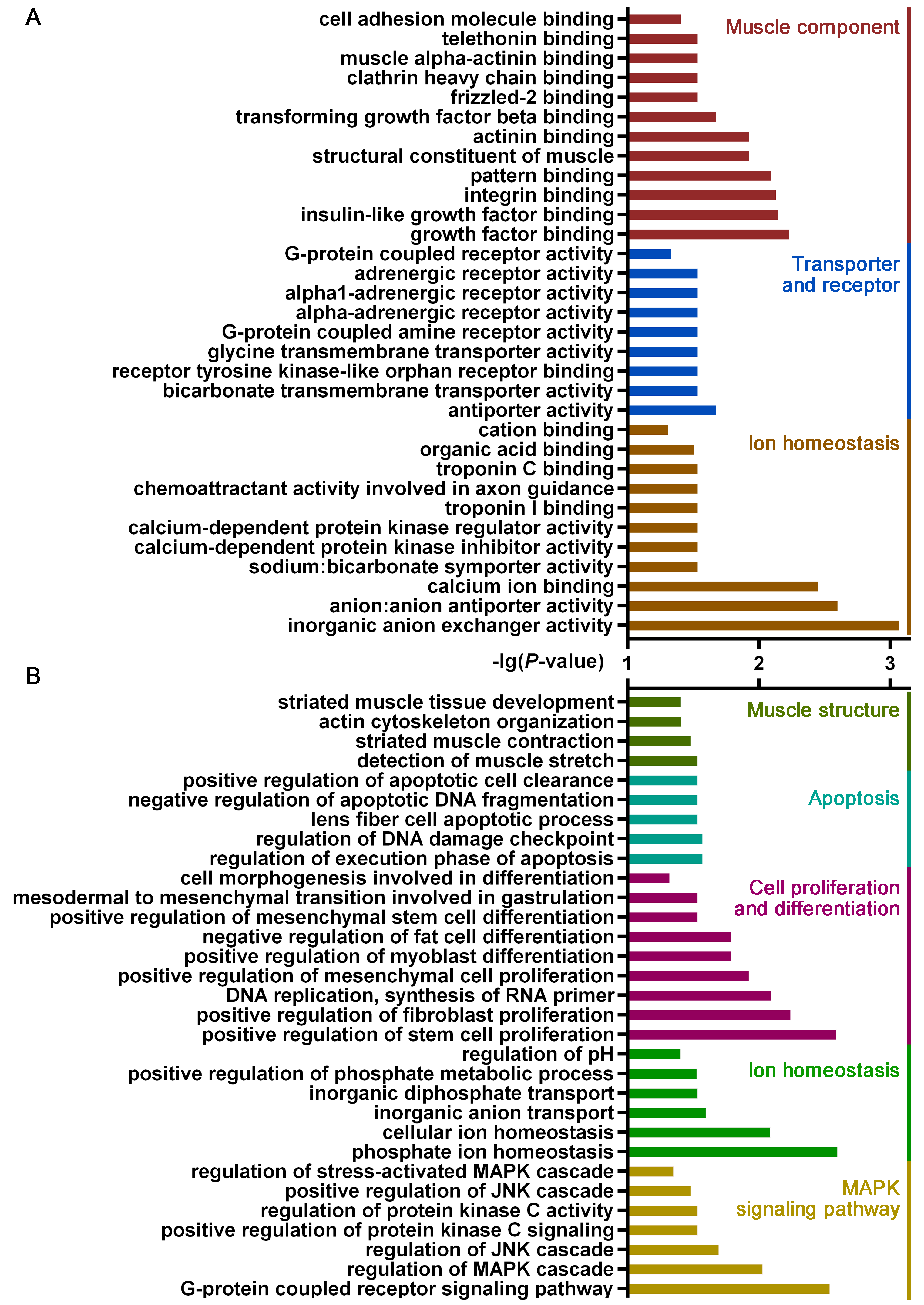

Supplement: Supplementary file 1 [file cells-09-01045-s001.zip › Figure S2.tif]

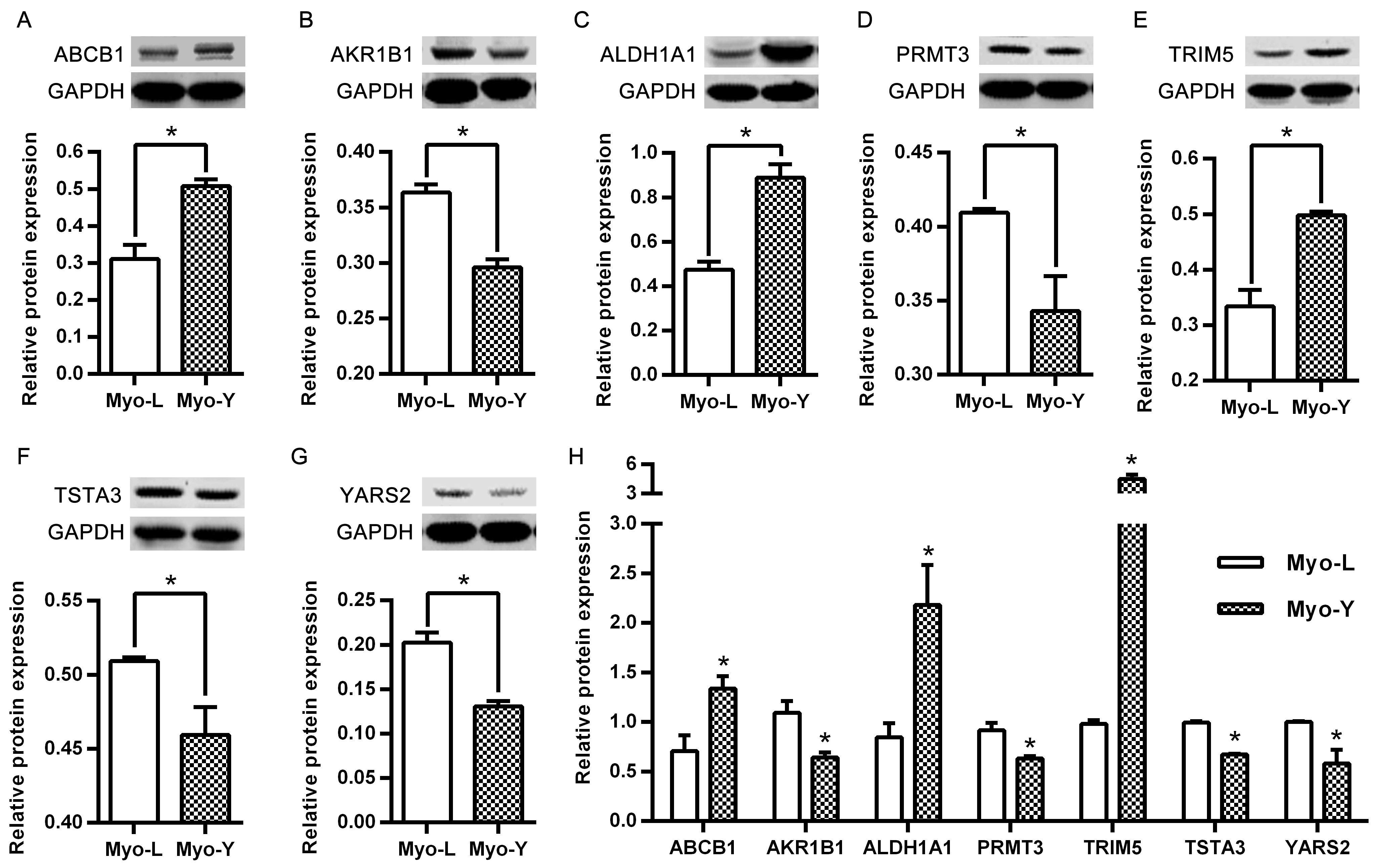

Supplement: Supplementary file 1 [file cells-09-01045-s001.zip › Figure S1.tif]

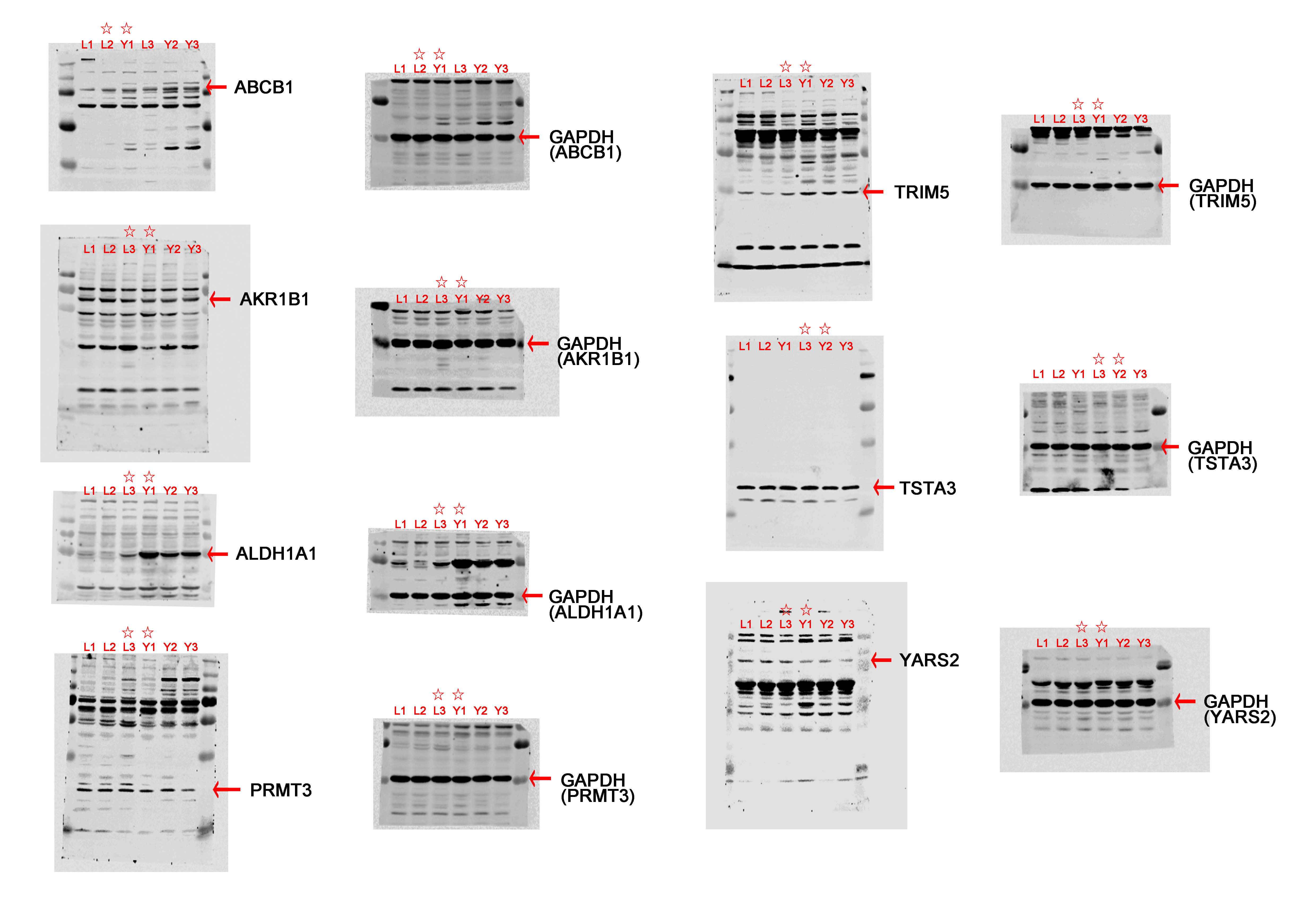

Supplement: Supplementary file 1 [file cells-09-01045-s001.zip › Raw data of Western Blot.tif]
